# Martial arts induce quasicritical brain states: a unified, multiscale, and mechanistic theory of brain health optimization

**DOI:** 10.3389/fpsyg.2025.1661566

**Published:** 2025-11-14

**Authors:** Caio Amaral Gabriel

**Affiliations:** Independent Researcher, São Paulo, Brazil

**Keywords:** martial arts, brain health, quasicriticality, neuroplasticity, metastability, functional connectivity, complex systems, heart rate variability

## Abstract

Emerging studies indicate that martial arts practice may benefit brain health; yet current findings are scattered and mostly descriptive, lacking an integrated explanation of underlying mechanisms. This article introduces the Integrative Theory of Martial Arts (ITMA), a theoretical framework developed through a systematic theoretical synthesis of interdisciplinary literature. ITMA explains how well designed martial arts training can optimize brain function by combining sensorimotor, cognitive and social-emotional demands. It proposes that these combined experiences drive the brain toward a quasicritical state—a dynamic regime of neural activity that supports health, efficient information processing, adaptability, and resilience. The theory integrates key concepts such as metastability, functional connectivity, neuroplasticity, neural synchronization, and signal-to-noise ratio as part of a single multiscale neurophysiological cascade. Preliminary empirical studies are cited to illustrate the plausibility of ITMA's propositions. By consolidating fragmented evidence into a unified mechanistic model, ITMA provides a new paradigm and testable hypotheses for future research on martial arts as a neurophysiological intervention, offering a roadmap for designing, evaluating, and scaling programs for brain health and human development.

## 1 Introduction

### 1.1 Context and rationale

Brain health underpins cognitive, sensory, social-emotional, behavioral and motor domains, enabling individuals not only to survive but also to thrive (World Health Organization, 2022; Damasio, 2018). It is also essential for achieving global agendas such as the Sustainable Development Goals (United Nations, 2015). Poor brain health entails severe individual, social and economic repercussions; thus, promoting brain health is both an end in itself and a critical enabler of other outcomes (World Health Organization, 2022).

In this context, martial arts represent a widely practiced, culturally embedded activity recognized in some cases as intangible cultural heritage (ICHCAP and ICM, 2020; ICM, 2021). Demonstrating their effectiveness for brain health could legitimize them as evidence-based, equitable, inclusive, and scalable non-pharmacological interventions. This would pave the way for low-cost public policies in health, education, and social development, with programs implementable in multiple contexts (communities, schools, hospitals, workplaces) and for various purposes—to prevent, treat, maintain, and promote brain health (Barry, 2019)—generating impact on both an individual and a global socioeconomic scale.

### 1.2 Research gap

Although a growing body of evidence suggests that martial arts practice benefits brain function (UNESCO and UNESCO-ICM, 2019; Vertonghen et al., 2014; Vertonghen and Theeboom, 2010; Jennings et al., 2022), recent reviews highlight that current findings remain scattered, predominantly descriptive, and lack a cohesive mechanistic framework (e.g., Ciaccioni et al., 2023). This fragmentation has two main dimensions:

**Conceptual dimension:** Most studies report psychological or social benefits without linking them to underlying neurophysiological pathways. Promising markers such as changes in BDNF or heart rate variability are often examined in isolation, without integration into a multiscale model explaining how these processes interact to drive system-level brain changes.

**Methodological dimension:** Studies vary widely in martial arts styles, training protocols, and participant experience levels, making it difficult to identify active ingredients or perform robust meta-analyses. Most research uses passive controls or no control group, preventing the disentanglement of effects specific to martial arts from those of general physical or social activity. There is no consensus on which neurophysiological outcomes or protocols to use, severely limiting comparability across studies. Longitudinal and randomized experimental designs with repeated measurements are rare, hindering causal inference and the understanding of long-term effects. Data are seldom integrated across behavioral, autonomic, neuroimaging, and molecular levels, limiting the potential for multiscale modeling.

Together, these limitations restrict understanding of causal pathways, the development of personalized programs, and the advancement of martial arts as an evidence-based field. The literature therefore lacks a unified, multiscale, and mechanistic model with explanatory and predictive power to articulate how martial arts training experiences translate into outcomes from the molecular to the behavioral level (Spaaij and Schaillée, 2020; Illari and Williamson, 2012; Funnell and Rogers, 2011; Bowman, 2019).

### 1.3 Objectives of this article

To address this need, this article aims to (1) propose the Integrative Theory of Martial Arts (ITMA) as a unified, mechanistic framework explaining how martial arts can optimize brain health; (2) consolidate scattered concepts into a multiscale neurophysiological cascade centered on quasicricality—a dynamic regime of neural activity that supports health, efficient information processing, adaptability, and resilience (Beggs, 2022; Williams-García et al., 2014); and (3) outline testable hypothesis and methodological innovations to guide future empirical research.

### 1.4 Positioning of martial arts

The ITMA does not claim that martial arts are the only activities capable of inducing quasicriticality. Rather, it presents them as a rich and condensed model for studying how embodied, social, and environmental components can converge to drive the brain toward optimal dynamics. Other practices and interventions may share similar mechanisms, but martial arts offer a coherent, culturally sustained structure in which these mechanisms can be systematically investigated.

### 1.5 Theoretical foundations

The development of ITMA was grounded in a systematic theoretical synthesis designed to integrate dispersed knowledge while maintaining epistemological rigor, conceptual soundness, and practical applicability. Rather than aggregating data empirically, this approach selected and combined mechanisms and concepts from multiple literatures according to explicit criteria and guiding principles:

**Interdisciplinarity:** The theory integrates knowledge from multiple disciplines to overcome the limits of single-discipline explanations of complex phenomena (Hampton and Parker, 2011; Carpenter et al., 2009; Akasofu, 2007; Bowman, 2019, 2015; Whitley et al., 2022; Stokols et al., 2013; Woods et al., 2021).

**Heterarchical thinking:** Concepts from different fields were treated as potentially equivalent and complementary rather than arranged in a strict hierarchy, avoiding reductionism that would obscure multiscale interactions (Berntson and Cacioppo, 2003).

**Consilience:** Preference was given to mechanisms supported by convergent evidence from independent fields, thereby increasing robustness and generalizability (Wilson, 1998).

**Cultural sensitivity:** The historical, political, and sociocultural particularities of martial arts were treated as constitutive rather than merely contextual, ensuring the theory respects the diversity of martial traditions (ICM, 2021; Foronda, 2008).

**First-Principles Reasoning:** The theory is grounded in the identification of universal mechanisms that explain how different martial practices, despite their specificities, can converge to optimize brain health (Tovstiga, 2023).

**Epistemological dynamism:** ITMA is conceived as an evolving construct, open to revision as new empirical evidence emerges, thus retaining theoretical relevance and methodological rigor over time (Capra and Luisi, 2014).

Concepts and mechanisms were included when they met all of the following conditions: (1) Direct relevance for explaining the neurophysiological effects of martial arts practices; (2) prior empirical support, even if preliminary, in complexity science, neuroscience, or related disciplines; and (3) potential for multiscale integration, linking molecular, neural, autonomic, behavioral and social levels. Conversely, concepts were excluded when they: (1) were purely speculative with no minimal empirical basis; (2) had relevance restricted to very specific contexts not generalizable to martial arts training; and (3) were redundant with mechanisms already incorporated.

**Figure 1:**
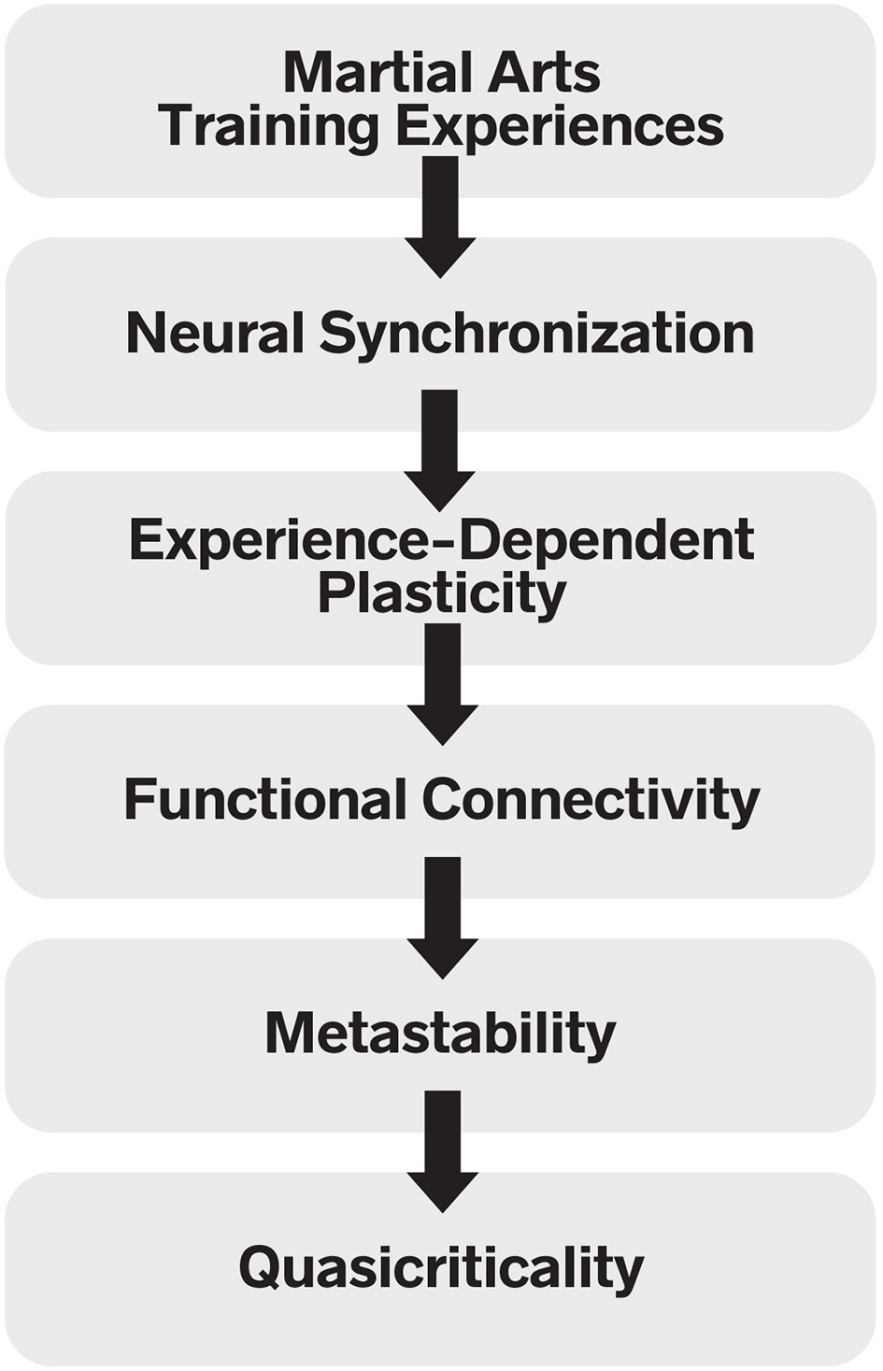
Integrative Theory of Martial Arts Mechanistic Cascade. The diagram illustrates the interactive flow of mechanisms proposed by ITMA, linking martial arts training experiences to optimal brain function. Starting with Neural Synchronization induced by specific embodied, relational, and environmental inputs, these patterns promote Experience-Dependent Plasticity, leading to lasting changes in Functional Connectivity. These changes, in turn, sustain Metastability, the dynamic balance between neural segregation and integration. Ultimately, metastability creates conditions for the emergence and maintenance of Quasicriticality, the brain's regime of maximum operational efficiency and adaptability.

The ITMA proposes a novel mechanistic cascade that links specific embodied, relational and environmental inputs of martial arts practice to downstream neurophysiological changes, ultimately promoting optimal brain function. As illustrated in Figure 1, according to ITMA, martial arts training experiences induces patterns of neural synchronization that, with repetition, promote experience-dependent synaptic plasticity and lasting changes in functional connectivity. These changes sustain metastability—the dynamic balance between segregation and integration—which, in turn, creates conditions for the emergence and maintenance of quasicriticality, the regime of maximum operational efficiency in complex systems such as the brain (Beggs, 2022).

A core methodological innovation of ITMA lies in its proposal of a functional triad of heart rate variability (HRV) as a pragmatic and ecologically valid set of proxies for brain quasicriticality, addressing the challenges of direct neuroimaging in real-world training environments. Specifically, this triad comprises measures of non-linear complexity (such as Detrended Fluctuation Analysis alpha 1 - DFA α1, and Sample Entropy - SampEn), alongside the sympatho-vagal balance (SD2/SD1 ratio from Poincaré plots). This triad—and not isolated HRV parameters—serves as a key outcome measure for evaluating the theory's predictions.

### 1.6 Structure of the article

The remainder of this article is organized as follows:

1. **Complexity science foundations**. The core concepts of complexity science are introduced as the fundamental theoretical basis for understanding ITMA.
2. **Mechanistic cascade of ITMA:** Using a “backward-thinking” approach, the article starts from the desired outcome—quasicriticality—and traces its necessary preconditions (metastability, functional connectivity, experience-dependent plasticity, neural synchronization, and signal-to-noise ratio). Each concept is explained in turn, and its specific implications for martial arts are discussed.
3. **Preliminary empirical illustrations:** In particular, subsections 5.6 and 5.8 review preliminary empirical evidence illustrating the plausibility of ITMA's propositions and addressing inter-individual variability in outcomes.
4. **Methodological innovations**: new tools are presented for evaluating martial arts interventions (e.g., non-linear HRV metrics, *N* = 1 designs, Bayesian translational approaches) that operationalize the theory's predictions.
5. **Implications and future directions:** The article concludes with recommendations for future research and a discussion of broader implications for policy, education, and practice.

### 1.7 Main testable hypothesis derived from ITMA

Drawing from the core propositions of the ITMA, the following testable hypotheses are advanced to guide future empirical research and validate the theory's mechanistic cascade and translational potential:

**Hypothesis 1—The core proposition (training induces quasicriticality):** a long-term martial arts intervention, designed based on ITMA principles (e.g., optimizing signal-to-noise ratio and neuroplasticity principles), will lead to a significant convergence of the heart rate variability (HRV) triad markers (DFA α1 approaching 1.0, increased SampEn, and an increased SD2/SD1 ratio) toward the quasicritical zone, detectable through methods that allow for the aggregation of individual trajectories (e.g., Bayesian *N* = 1 meta-analysis or heterogeneity-preserving pragmatic trials), when compared to an active control group.

**Hypothesis 2—The proxy validation (HRV correlates with brain dynamics):** changes in the HRV functional triad markers will be positively correlated with direct neuroimaging markers of metastability and quasicriticality (derived from fMRI or EEG) in the same individuals.

**Hypothesis 3—The neurophysiology-behavior correlation (functional relevance):** the magnitude of changes toward quasicriticality, as measured by the HRV functional triad, will be positively correlated with the magnitude of improvements in specific functional outcomes that were the target of the intervention. For example, in a martial arts program designed to enhance attentional control, improvements in attention tests (cognitive domain) will correlate with positive changes in HRV markers. Similarly, in a program focused in emotional regulation, improvements in measures of emotional reactivity (social-emotional domain) will also correlate with changes in HRV.

**Hypothesis 4—The modulation by signal-to-noise ratio (quality of experience):** individuals participating in a martial arts program with a high signal-to-noise ratio—resulting from an intentional design based on neuroplasticity principles—will exhibit greater gains in quasicriticality markers compared to individuals training with the same technical content in an environment with a low signal-to-noise ratio.

**Hypothesis 5—The neurodynamics of inputs (waves vs. vortices):** training tasks emphasizing global integration will predominantly induce “wave-like” neural dynamics (increased global functional connectivity). In contrast, training tasks emphasizing segregation and specialization will predominantly induce “vortex-like” dynamics (increased local connectivity and segregation of specific networks).

**Hypothesis 6—The prediction of individual responsiveness:** baseline measures of endogenous factors will significantly predict the magnitude of neurophysiological response (the shift toward quasicriticality) after a training period.

These hypotheses collectively provide a roadmap for empirical validation of ITMA's mechanistic cascade and translational potential.

## 2 Martial arts as modulators of neural dynamics

### 2.1 The brain as a complex system

The explanatory power of ITMA lies in its foundation in complex systems science. This first-principles approach allows the theory to transcend the technical and cultural particularities of each martial art to focus on the fundamental mechanisms that govern neural dynamics. By understanding the brain as a complex system guided by principles of emergence and self-organization (Kelso, 1995; Bak, 1996), ITMA can then explain how such diverse practices can converge to induce the same outcome: the optimization of brain health.

A system is a set of elements that interact with each other (Von Bertalanffy, 1968; Meadows, 2008). The human brain is an example, composed of approximately 86 billion neurons and various non-neuronal cells (e.g., glial cells; Azevedo et al., 2009). Its components interact through synapses, where electrochemical signals are transmitted. Glial cells, in turn, not only support neurons but also actively modulate neural communication, enabling the coordinated functioning of the brain (Kandel et al., 2021; DeFelipe et al., 2002).

The brain, however, is a complex system, with characteristics that allow it to perform extraordinary feats (Kelso, 1995; Pessoa, 2022; Bullmore and Sporns, 2009). Although there is no universal definition, complex systems are typically understood as a set of elements whose interaction produces emergent properties (Ladyman and Wiesner, 2020). With its vast and intertwined network of connections, the brain is considered one of the most complex systems known (Green et al., 1998), manifesting unique characteristics that arise irreducibly from the interactions among its elements (Meadows, 2008).

### 2.2 Emergence and self-organization

An emergent property is a phenomenon that arises solely from the interaction among the elements of a system. Emotions, cognition, and behavior, for example, are emergent properties that cannot be explained by isolated neurons, reflecting the principle of synergy, where the whole is greater than the sum of its parts (Waldrop, 2019; Jensen, 2022).

However, emergence is not a static event but a continuous process, as the brain is inherently dynamic. Its dynamics are characterized by “fluctuations”—coordinated and structured variations in neural activity that continuously reorganize across multiple scales. Change, therefore, is not the exception but the rule of its functioning (Northoff, 2024; Pessoa, 2022). It is from this dynamic flow that phenomena emerge moment to moment and the brain's complex functions are sustained.

Among the emergent properties, self-organization stands out: the capacity of a complex system to monitor and modify its own activity spontaneously, continuously, and dynamically, without central control or external instructions. Functionally, the brain exhibits autonomy, regulating itself without depending on external commands (Kelso, 1995).

### 2.3 Neural negentropy

Self-organization is the dynamic output that emerges from the synergistic interaction of multiple inputs, which shape neural dynamics in real-time (Northoff, 2024). This raw material for self-organization includes: (1) the brain's intrinsic activity (neural noise), a dynamic baseline generated even in the absence of external stimuli; (2) interoception, which informs the brain about the body's internal state (e.g., heartbeat, visceral signals, microbiota); and (3) external stimuli, as the brain, being an open system, aligns its activity with social and environmental stimuli through specialized neural systems (e.g., the social engagement system, mirror neurons, theory of mind; Porges, 2011; Rizzolatti and Sinigaglia, 2010; Frith and Frith, 2005; Northoff, 2024).

The function of this self-organization is adaptive: to counteract the tendency toward entropy (disorder) described by the second law of thermodynamics for closed systems (Prigogine and Stengers, 1984). As living, open systems, organisms depend on autopoiesis (self-maintenance), a process that requires negentropy—a flow of organized energy and information used by the system to maintain order and avoid collapse (Maturana and Varela, 1980; Schrödinger, 1944). In the brain, self-organization is the autopoietic process that utilizes internal noise, interoception, and external interactions as negentropic raw material, creating the flow of energy and information necessary not only for survival but also for the individual's prosperity (Damasio, 2018).

If self-organization is what the brain does, the predictive model explains how. To survive and thrive, the brain must anticipate, not just react. It acts as an active inferential system, generating predictions about the future by continuously integrating the negentropy of past experiences with present signals. Therefore, self-organization is not random; it is guided by hypotheses that aim to reduce uncertainty and prediction error, thereby optimizing decisions and actions (Friston, 2010; Friston et al., 2017). This predictive mechanism ensures survival by anticipating threats and enables prosperity by projecting opportunities for learning and wellbeing.

### 2.4 Neural plasticity and martial experiences

A key implication of self-organization is that the brain is profoundly shaped by experience, in a process where nature and nurture act as complementary forces in neurodevelopment (LeDoux, 2002). Experience actively modulates neural dynamics; neurodynamically, it corresponds to the coordinated activation of neural pathways in response to internal and external stimuli. Crucially, the effect of an experience depends not only on the stimulus itself, but also on the dynamic state of the brain at that moment.

When experiences are systematized, they can induce lasting changes in the brain's architecture through neuroplasticity—the brain's ability to adapt by modifying its structures, functions, and connections (Cramer et al., 2011). This is the fundamental biological mechanism for learning and continuous development. In this context, martial arts stand out as an intentional intervention to reorganize brain dynamics. By acting directly on the inputs of self-organization (intrinsic activity, interoception, social interactions, and environmental stimuli), martial training experiences can constitute an exceptionally dense and high-quality source of essential negentropy.

The goal of training, therefore, is to induce patterns of neural activity that refine synaptic interactions. This drives a more functional self-organization that sustains a healthier, more efficient, adaptable, and resilient brain—a brain prepared not only to survive, but to thrive.

### 2.5 Health, disease, and healing as dynamic processes

Complex systems science posits that health, disease, and healing are not fixed states, but dynamic processes. As expressions of emergence, self-organization, and plasticity, these processes can be influenced by systematic experiences, like martial arts practice, which optimize neural interactions and drive a functional self-organization to promote brain health (Northoff, 2024; Naviaux, 2018, 2023; Siegel, 2020).

This raises fundamental questions: What defines this “functional self-organization”? What is the ideal dynamic state of a healthy brain? And, neurobiologically, what is the primary target that martial arts practice should aim for to optimize brain health?

## 3 Quasicriticality: the organizing principle of brain health

### 3.1 Definition and evidence of quasicriticality

The central proposition of ITMA is that martial arts optimize brain health by inducing a quasicritical state: a dynamic regime of neural activity that maximizes the brain's health, information processing, adaptive capacity, and resilience to perturbations (Beggs, 2022; Williams-García et al., 2014).

Empirical evidence supports this concept, with quasicriticality being observed in the resting-state brain activity of healthy individuals and in simulations of neuronal cultures and cortical slices (Fosque et al., 2022, 2021). Consequently, quasicriticality is proposed as a biomarker (Fosque et al., 2022) and predictor (Beggs, 2022) of brain health. In contrast, deviations from this state are associated with impaired brain health (Zimmern, 2020), such as epilepsy (Meisel et al., 2012), Alzheimer's disease (Montez et al., 2009), tauopathy (McGregor et al., 2024), depression (Gärtner et al., 2017), and sleep deprivation (Meisel et al., 2013).

The phase transition of water (e.g., from liquid to ice or vapor) serves as an analogy (Kauffman, 1995). Just as water completely reorganizes upon reaching certain thresholds, neural activity can organize into distinct dynamic regimes, each with its own characteristics and different impacts on brain health (Kauffman, 1995).

### 3.2 The river metaphor: navigating between order and chaos

**Figure 2:**
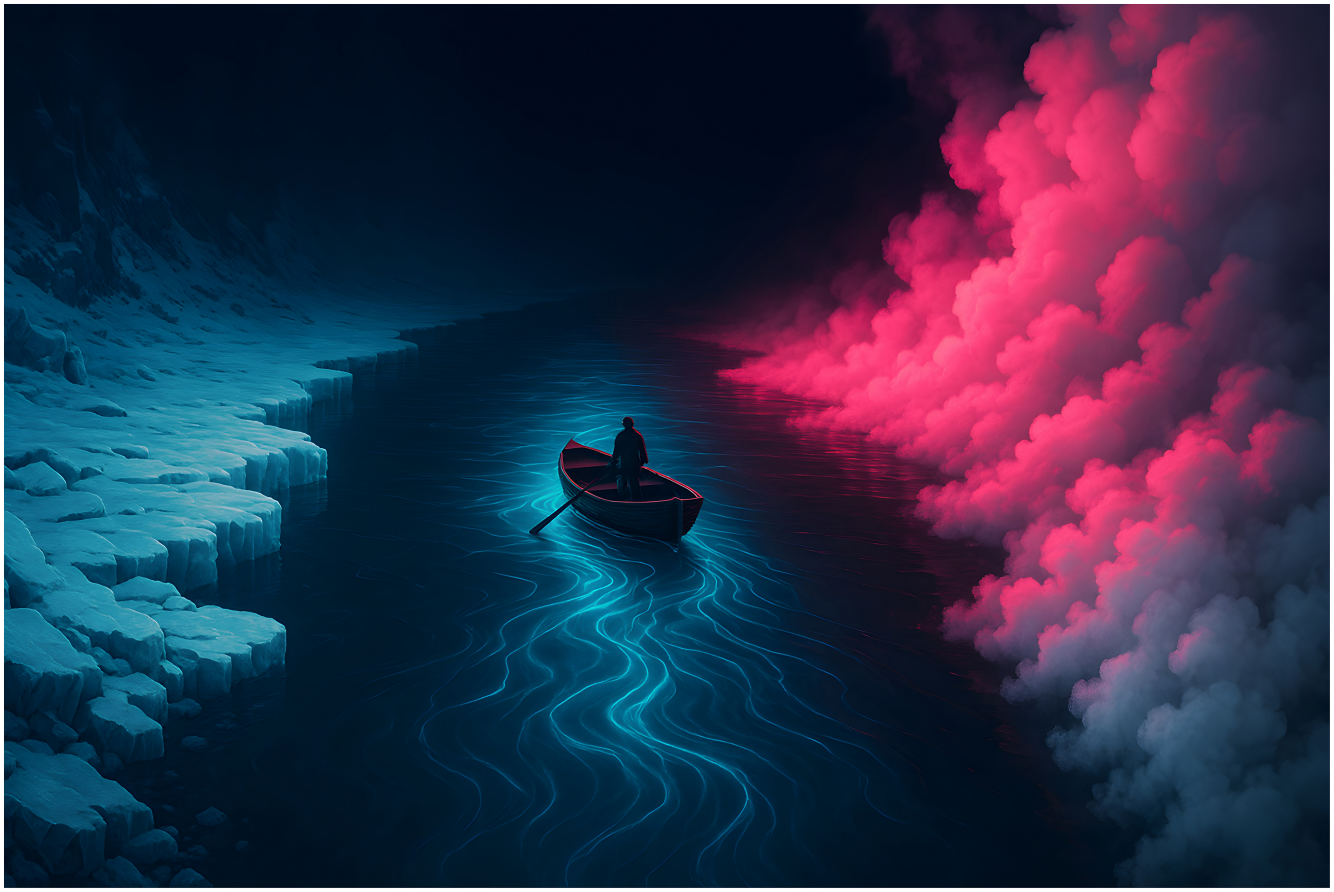
The river as a metaphor for quasicriticality. On the left, the frozen bank symbolizes the excessive order (subcriticality). On the right, the boiling bank symbolizes the excessive chaos (supercriticality). The central flow represents the ideal state of quasicriticality, which the practitioner navigates to achieve brain health, efficiency, adaptability and resilience.

The metaphor of a river can illustrate quasicriticality and its deviations (see Figure 2). The central flow of the river, where the water runs adaptively, represents the quasicritical state. This is the brain's ideal regime, characterized by an enhanced capacity for complexity and adaptation. In this condition, the brain modulates its activity patterns in a flexible and efficient manner, improves communication between regions—even distant ones—and exhibits greater robustness to perturbations. A healthy brain is like a boat that skillfully navigates this flow, maintaining its functional integrity (Fosque et al., 2022; Shew and Plenz, 2012; Helias, 2021).

However, internal and external stimuli can push the boat toward the banks, which represent dysfunctional dynamic states. On one side, the frozen bank symbolizes subcriticality, a regime of excessive order, where neural activity becomes rigid, predictable, and repetitive, like ice. On the other, the boiling bank symbolizes supercriticality, a regime of excessive chaos, where neural activity is highly turbulent, unpredictable, and random, like steam. The conceptual boundary that separates the healthy flow from these dysfunctional banks is the “Widom line”, a functional threshold that, if crossed, impairs the brain's adaptive capacity. The objective, therefore, is to keep the brain's dynamics navigating in the quasicritical region, in a functional equilibrium between the extremes of order and chaos, preventing the system from collapsing into one of these states (Beggs, 2022; Fosque et al., 2022).

### 3.3 The drive toward quasicriticality: development and salugenesis

The brain has evolved intrinsic mechanisms that enable it to approach, maintain, and return to the quasicritical regime—a dynamic attractor state that maximizes both stability and adaptability. This natural convergence reflects a phenomenon known as self-organized quasicriticality (Bak, 1996; Kinouchi et al., 2020), wherein the system spontaneously tunes itself to a functional edge between order and chaos. This attractor is not static but is sustained through developmental plasticity and allostatic regulation, which continuously recalibrate the system in response to internal and external demands. From an evolutionary standpoint, quasicriticality represents the culmination of a billion-year process of optimizing resilience: the capacity to remain functionally coherent while flexibly reorganizing in the face of perturbations (Aboitiz, 2024).

Neurodevelopment can be understood as a dynamic trajectory in which the brain “learns” to self-organize toward quasicriticality. This journey begins in neurulation and the early stages of neurogenesis, with the brain operating in a state of low activity and connectivity. As neurogenesis intensifies and synapses are overproduced, the system swings to one extreme: a supercritical regime of high excitability. Subsequently, a series of refinement processes—including neuronal migration, differentiation, and, crucially, synaptic pruning that eliminates redundant connections—cause the system to swing to the opposite extreme: a more ordered, subcritical regime. Finally, with the advancement of myelination and the establishment of firing rate homeostasis, which optimizes the balance between excitation and inhibition, the brain stabilizes in the quasicritical regime. This final state, functionally more stable and adaptive, is the result of this developmental calibration that transitions between the “chaos” of creation and the “order” of refinement (Kielty et al., 2024; Tetzlaff et al., 2010; Beggs, 2022; Young, 2022).

Following perturbations, allostatic processes act to maintain or return the brain to the quasicritical region (Ma et al., 2019; Fontenele et al., 2019; Shew et al., 2015; Tetzlaff et al., 2010). ITMA proposes that salugenesis is the central biological process that drives this return. Primarily mediated by mitochondrial activity, salugenesis unfolds in three phases: (1) inflammation, a defensive response to contain damage; (2) proliferation, a phase of reconstruction and biomass replacement; and (3) differentiation, a phase of remodeling and maturation that results in a more resilient systemic organization. The successful completion of this sequence re-establishes quasicritical dynamics (Naviaux, 2018, 2023, 2014).

However, if allostatic load (intense and/or prolonged stimuli) exceeds the system's regulatory capacity, or if the phases of salugenesis are blocked, the brain loses its ability to return to its healthy attractor. In the river metaphor, the boat becomes stranded on the dysfunctional banks of sub- or supercriticality. Sustained neural dysfunction, therefore, does not arise from the deviation itself, but from the system's failure to return to the quasicritical flow after the perturbation (Shew et al., 2015; Naviaux, 2018, 2023; Beggs, 2022; Sterling, 2020).

### 3.4 Implications of quasicriticality for martial arts

Research on quasicriticality establishes that the brain operates in this regime to optimize its function, that it self-organizes toward this state, and that deviations from it imply dysfunction. These findings generate profound implications for martial arts programs:

**Quasicriticality as a universal target:** ITMA proposes that quasicriticality is the ultimate neurobiological goal of any training program focused on brain health, regardless of the martial tradition constituting the program. This allows for theoretical unification without practical standardization: each tradition may have distinct methods, but they converge toward the same optimal functional state.

**Direction over origin:** regardless of a practitioner's initial conditions or specific demands, the path to optimization converges on a single neural destination: guiding the brain's dynamics toward quasicriticality.

**New functional definitions:** the ITMA framework supports new functional definitions for health, brain health, and healing.

1. Health is a living system's capacity to respond adaptively to perturbations, maintaining functional integrity by navigating the threshold between order and chaos to survive and thrive in a constantly changing world.
2. Brain health is the manifestation of quasicriticality in neural dynamics.
3. Healing is the process of self-organization, via successful salugenesis, that generates a new, more resilient and functional systemic organization (instead of the restoration of a previous state).

With this, the goal of training transcends the improvement of isolated skills and becomes the optimization of a neurodynamic state—quasicriticality. This allows both the design of interventions (the instructor's role) and the evaluation of their outcomes (the researcher's role) to be guided by a fundamental neurobiological principle, making the field more rigorous and effective.

**Dysfunction as deviation:** in ITMA, dysfunctions in a practitioner's domains of brain health—cognitive, sensory, social-emotional, behavioral, and motor—are reinterpreted as one of three patterns of deviation from quasicriticality: excessive order (subcriticality), excessive chaos (supercriticality), or a mixed pattern. Crucially, these dynamic deviations manifest as impairments in the brain's functional connectivity.

**Disease as collapse into a pathological attractor:** from the perspective of ITMA, disease can be functionally defined as the process by which a living system collapses and stabilizes into a pathological attractor of excessive order (subcriticality) and/or excessive chaos (supercriticality). This collapse can occur via three main pathways: (1) a failure to achieve quasicriticality, often resulting from deviations in the course of neurodevelopment, which prevent the consolidation of healthy dynamic patterns; (2) a failure to return to quasicriticality after perturbations, due to the blockage of the phases of salugenesis, compromising the system's adaptive reorganization capacity; and (3) a restriction imposed by senescence, wherein physiological aging progressively reduces the brain's complexity and functional variability. In all these scenarios, the result is the loss of the ability to achieve, maintain, or return to the quasicritical state, causing the brain to become chronically trapped in dysfunctional patterns of functional connectivity.

Crucially, in this perspective, the term “pathological” does not refer to a deviation from a statistical norm or an idealized “normal brain”—a concept that is untenable in complex systems. Dysfunction, in the context of ITMA, is defined by the loss of adaptive capacity and the suffering that emerges when the system becomes chronically trapped in these states of excessive chaos and/or order. Neurodiversity is understood here as the existence of multiple valid brain architectures and developmental trajectories. In this sense, the goal of martial arts training is not to “normalize” a neurodivergent brain, but rather to provide the inputs that allow any type of brain to optimize its own self-organization, manage its allostatic load, and find its way back to its optimal zone of functioning—quasicriticality.

**Influencing change to achieve stability:** martial arts practice does not “change” a brain that is already naturally in constant transformation but allows for the intentional influencing of this reorganization's direction. The goal, counter-intuitively, is not change itself, but stability in the quasicritical region. The implication of this for practice and research is profound: the success of an intervention or program is no longer measured by gains in isolated parameters, but by the optimization of the system's dynamic capacity to achieve, maintain, and return to its ideal functional regime.

**Neurodevelopmental optimization:** martial arts can be adapted as early interventions to guide the brain's developmental trajectory and neural maturation toward quasicriticality, promoting a healthier neuro-ontogeny.

**Transformation, not restoration:** aligned with salugenesis, martial arts practice does not “restore” a previous state but promotes the emergence of a new, more functional and adaptive neural organization.

**A continuous journey:** the optimization of quasicriticality is not a final destination but a dynamic process of constant maintenance throughout life, requiring deliberate engagement.

But what operational principle allows the brain to sustain quasicriticality moment to moment, navigating the constant fluctuations of life without collapsing into patterns of excessive order or chaos?

## 4 Metastability as a precondition for quasicriticality

### 4.1 The paradox of neural adaptation

The adaptive brain faces a paradoxical challenge: it must be flexible enough to respond to a changing world, yet stable enough to maintain functional cohesion without collapsing into disorder. An excessively rigid or unstable brain becomes dysfunctional. In the river metaphor, the boat must adjust its course (flexibility) without losing control and sinking (stability). The mechanism that dialectically resolves this tension, allowing the brain to be both flexible and stable at the same time, is metastability (Tognoli and Kelso, 2014).

### 4.2 Segregation and integration: the pillars of metastability

Metastability is the dynamic equilibrium between two complementary and simultaneous processes: segregation and integration. Segregation refers to the specialization and autonomy of neural regions, while integration describes the coordination and synergy among them (Tognoli and Kelso, 2014). Metaphorically, metastability is like a jazz ensemble: each musician improvises with a unique voice (segregation), but the performance only becomes harmonious because they all follow a shared rhythmic and harmonic structure (integration).

Crucially, segregation and integration are contextually flexible processes. According to the concept of entanglement (Pessoa, 2022), a single neural structure can assume different functional roles by participating in multiple networks that form, overlap, and dissipate dynamically. The jazz musician, for example, can be a soloist at one moment and a rhythm player the next, depending on what the music demands. This continuous alternation between the transient autonomy of networks (segregation) and their flexible coordination (integration) allows the brain to transition among a myriad of neural configurations, thus solving the flexibility-stability paradox.

### 4.3 Neural basis of metastability

The equilibrium between segregation and integration is made possible by the brain's architecture, which has been optimized throughout evolution to resolve this paradox (Sterling and Laughlin, 2015).

The map describing this architecture is the connectome: the complete set of brain regions and their connecting pathways (Sporns, 2012; Seung, 2012). Morphologically, segregation is manifested in the physical separation of the brain into distinct modules (e.g., lobes, nuclei), while integration is ensured by white matter tracts that connect them and by cortical folding (gyri and sulci), which optimizes the length and cost of the neural “wiring”. However, the architecture's efficiency lies in its topology—the abstract pattern of its connections. From a graph theory perspective, the connectome is a “small-world” network, combining high local clustering (the basis of segregation) with short global paths (the basis of integration). The network is also “scale-free”, characterized by the presence of hubs—highly connected nodes that act as communication centers, efficiently facilitating global coordination. This inseparable union of efficient morphology and topology sustains metastability, creating the conditions for the brain to operate in the quasicritical state (Barabási, 2014; Sporns, 2016).

### 4.4 The functional dynamics of a healthy brain

To understand metastability, one must distinguish the brain's physical architecture from its real-time activity. While structural connectivity (the connectome) describes the anatomical pathways, functional connectivity describes the dynamic flow of neural activity along these pathways. It is defined as the temporal correlation between the activity of different brain regions—regardless of whether they are physically linked—revealing which of the brain's “musicians” are “playing together” to form transient and adaptive functional networks (Pessoa, 2022; Sporns, 2016).

The processes of segregation and integration are, in effect, observable patterns of functional connectivity. Segregation occurs when there is high functional connectivity within a network but low connectivity with the rest of the brain, creating a “vortex” of specialized neural activity. On the other hand, integration manifests as an increase in functional connectivity among multiple modules and hubs, generating “waves” of synchronized activity that propagate throughout the brain to coordinate global responses (Deco et al., 2024, 2023; Deco and Kringelbach, 2020; Martínez-Molina et al., 2024; Escrichs et al., 2022, 2024). Percolation, a fundamental principle of complex systems, describes how this transition from a local to a global state occurs: large-scale communication only emerges when the network's connectivity reaches a critical threshold. By operating near this threshold, the metastable brain can fluidly alternate between regimes of segregation and integration (Broadbent and Hammersley, 1957; Tian and Sun, 2022).

### 4.5 When metastability fails

When metastability fails, the brain is deviated from the quasicritical region (Lord et al., 2017). Impairments in functional connectivity can lead to an imbalance, resulting in dysfunctional regimes. Excessive integration at the expense of segregation leads to a rigid order—the subcritical regime—like a monotonous band playing the same note. Conversely, excessive segregation at the expense of integration leads to chaos—the supercritical regime—like a dissonant cacophony. Thus, brain health, like good music, resides in the dynamic equilibrium between the autonomy of the parts and the coherence of the whole (Tognoli and Kelso, 2014).

### 4.6 Empirical evidence of metastability

Empirical studies corroborate metastability as a crucial marker of brain health (Deco et al., 2017, 2015). Cognitive flexibility and metastability are reduced in cases of traumatic brain injury (Hellyer et al., 2015), an impairment linked to maladaptive processes such as diaschisis—in which damage to one area affects the function of other distant but connected regions—and transneuronal degeneration—the atrophy of neurons due to the loss of connection with the injured area (Fornito et al., 2015). In healthy individuals, greater neural flexibility—more transitions between functional connectivity states—correlates with better executive performance (Nomi et al., 2017). More broadly, impaired metastability is a common feature of multiple disorders (neurodevelopmental, neuropsychiatric, and neurodegenerative), which may reflect a deviation from quasicriticality and a process of network dedifferentiation—a process in which neural networks lose their functional specialization, becoming less distinct and efficient (Lord et al., 2017; Fornito et al., 2015).

The structure of the connectome itself also supports this view. A study of 461 healthy adults demonstrated that a more interconnected connectome was associated with favorable indicators such as fluid intelligence, better cognitive performance, and life satisfaction. In contrast, a less interconnected connectome correlated with unfavorable traits such as substance use and anger. Therefore, connectome interconnectivity can explain individual differences in brain health, serving as a structural substrate that sustains metastability and, consequently, quasicriticality in the brain (Smith et al., 2015).

These dynamic and structural pieces of evidence support that metastability is vital for healthy brain functioning and that its impairment is a common denominator in multiple pathologies. Crucially, this positions the re-establishment of metastability as a central, evidence-based therapeutic target for health interventions (Lord et al., 2017).

### 4.7 Implications of metastability for martial arts

Empirical evidence supports that metastability is vital for brain health, that its impairment is associated with multiple disorders, and that it can be optimized by interventions. These findings generate the following implications for martial arts training programs:

**Optimizing the segregation-integration balance**: ITMA proposes that martial arts can optimize metastability by re-establishing the functional balance between the specialization and coordination of neural networks—integrating regions that should be more connected and coordinated, and segregating regions that should be more differentiated and independent—thus removing obstacles to the brain's natural tendency to self-organize toward health.

**A dynamic taxonomy of disorders:** brain dysfunctions can be classified based on their patterns of metastability failure—excessive order (subcritical), excessive chaos (supercritical), or a combination of both. This taxonomy for brain health impairments, based on dynamic processes, can complement traditional diagnostic models and guide the design of personalized interventions.

**Intentional design for metastability:** training sessions can be intentionally designed with the pedagogical and andragogical goal of optimizing functional connectivity and, consequently, metastability.

**Neurodynamic classification of interventions:** ITMA proposes that the interventions composing the training experiences can be functionally classified based on the predominant type of neural activity pattern they evoke in the system:

1. “Wave-like” dynamics: characterized by the coordinated and coherent propagation of neural activity across different regions, promoting functional integration and global synchrony. Interventions with this pattern are ideal for strengthening hubs, consolidating coordination between networks, and fostering states of functional cohesion.
2. “Vortex-like” dynamics: characterized by localized, oscillatory, or recursive activation in specific regions, promoting functional differentiation and the selective segregation of networks.

The intentional and alternating application of these two patterns allows not only for the promotion of metastability, but also for the personalization of training experiences according to the practitioner's functional profile and goals. This introduces a new operational principle: the design of neurodynamic experiences based on the induced oscillatory pattern.

**Focus on contextual dynamics, not localization:** in light of the concept of entanglement, the description of effects must abandon the simplistic model “martial art A improves neural network B, associated with function C” and adopt a dynamic framework: “Intervention A, in context B, favors the emergence of neural pattern C”. This perspective requires both research and practice in martial arts to consider not just the intervention itself, but the entire context in which it is practiced, as the context defines the resulting neurodynamic pattern.

**“Hub training” for resilience:** martial arts, by requiring the rapid integration of multiple systems, can act as a form of “hub training”, strengthening the central nodes of the brain's network and increasing its robustness against perturbations.

**Promoting a robust connectome:** the continued practice of martial arts can strengthen the neural network's infrastructure itself, promoting a more interconnected connectome. This aligns with findings that associate greater connectivity with indicators of health and wellbeing, thereby increasing the brain's capacity for metastability.

**Optimizing the “resilience triad”:** martial arts training experiences can optimize the brain's “resilience triad” (Fornito et al., 2015) through three distinct and complementary mechanisms:

1. Reserve is optimized by promoting a more robust and interconnected connectome, which increases the brain's passive capacity to withstand damage before symptoms manifest.
2. Degeneracy is trained through activities that teach multiple paths to the same end, creating structurally distinct but functionally equivalent neural pathways. This makes the system less fragile, as if one response pathway fails, the brain has a vast repertoire of trained alternatives to recruit.
3. Compensation is enhanced through experiences that intentionally promote metastability, optimizing the brain's active process of functional reorganization after damage.

This multifaceted optimization of resilience positions martial arts as preventive and potent proactive rehabilitative practices.

But if the optimization of functional connectivity is what maximizes metastability, what mechanisms sustain this optimization over time?

## 5 Plasticity and synchronization as the basis of metastability

### 5.1 Neural synchronization: the immediate mechanism of functional connectivity

ITMA proposes that martial arts training experiences can be designed to generate specific patterns of neural synchronization. The consistent repetition of these patterns strengthens functional connectivity, triggering synaptic plasticity that, in turn, “sculpts” the neural network to sustain metastability and induce quasicriticality. Neural synchronization is, therefore, the mechanism that enables functional connectivity in real time. Technically, it is the temporal coordination of the oscillations of neuronal populations, which aligns their phases or frequencies to promote integration and efficient communication between brain regions.

The biophysical substrate for synchronization is the intrinsic timescale of each brain region, which determines the degree of neural persistence—the capacity for a neuron's activity to continue over time after a stimulus has ended, functioning as a neuronal short-term memory. This persistence creates the temporal window that allows for the rhythmic alignment between different neuronal populations, a process that transiently strengthens the functional connectivity between the involved regions. In practice, for functional connectivity to be consolidated, neural regions need to be co-activated in a repeated, synchronized, and sustained manner (Northoff, 2024).

### 5.2 Experience-dependent synaptic plasticity: consolidating functional connectivity

If neural synchronization creates the transient conditions, experience-dependent synaptic plasticity is the biological process that consolidates them into lasting changes. The repetition of synchronized patterns activates plastic mechanisms that sculpt the brain's architecture in response to use—not globally, but precisely in the neural pathways and regions activated during the experience. The process of consolidating functional connectivity is governed by Hebb's Law: “neurons that fire together, wire together”. In practice, repeated training experiences bring synapses to a bifurcation point, a concept from complex systems that describes how a small change in neural activity can cause an abrupt shift. If the co-activation of networks reaches a critical threshold, the synapse strengthens via long-term potentiation (LTP); if the activity is desynchronized, it weakens via long-term depression (LTD). This process is governed by path dependence: each strengthening via LTP increases the probability of the same pathway being activated in the future. This cumulative effect, resulting from the dynamic competition between LTP and LTD, sculpts the network's architecture; therefore, martial arts training can be seen as an intentional process to guide this path dependence (Merzenich et al., 2014; Nahum et al., 2013).

However, the continuous repetition of the same stimulus leads to a phenomenon of adaptive neural suppression, in which the amplitude of the brain's response decreases as it learns to process information more efficiently (Northoff, 2024). Although this initial suppression is a functional marker of learning, its persistence without variation can result in neurodynamic stagnation, restricting new opportunities for plasticity. For the optimization process to continue, training must follow the principle of progression, analogous to “progressive overload” in physical training: experiences must be gradually and continuously challenging, introducing new, prediction-error-rich stimuli that force the brain out of its comfort zone and to reorganize. When this principle is well-applied, the system can enter a positive feedback loop—what (Fredrickson 2013) termed an “upward spiral”—in which small neurofunctional improvements facilitate richer experiences, which in turn induce further positive reorganizations. This upward spiral is the functional opposite of degenerative patterns and can sustain the system's progressive approach toward the quasicritical regime, promoting not only stability but also an expansion of adaptive capacity.

Crucially, neuroplastic changes triggered during wakefulness are consolidated and stabilized during sleep through complex processes involving synaptic renormalization, memory reactivation, and cortical reorganization—especially via the sequential interplay of SWS and REM sleep cycles (Gorgoni et al., 2013).

Moreover, changes in neural architecture obey a biologically embedded temporal pattern. Emerging evidence suggests that new gene expression patterns begin to consolidate within 3–4 days of consistent stimulation, while stable physiological reorganization may take about 3 weeks. Structural neural remodeling becomes measurable after approximately 3 months (Naviaux, 2018). These temporal dynamics, encoded by evolution, highlight the need for sustained and adaptive exposure over time to consolidate plastic gains and stabilize new functional regimes.

The beneficial effects induced by this process do not cease with the end of the stimulus. On the contrary, they tend to persist due to a fundamental principle called hysteresis: the capacity of a system to maintain the effects produced by an experience, even when it is no longer present (Ferraris, 2024). In the context of ITMA, this means that the well conducted practice of martial arts can generate durable neurofunctional changes, whose benefits remain even after temporary interruptions, thereby strengthening the system's long-term stability.

While many principles of neuroplasticity have been identified in the literature, ITMA proposes that five of them articulated in this section—specificity, repetition, progression, rest, and temporality—form the core operational cycle required to induce and consolidate functional neuroplastic change. Each plays a distinct role in the process: specificity determines the target of change; repetition initiates and strengthens the circuit; progression forces adaptive reorganization; rest consolidates the gains; and temporality structures the entire sequence.

### 5.3 Horizontal and vertical integration: the multidimensional architecture of connectivity

ITMA postulates that the patterns of connectivity and plasticity optimized by training are multidimensional, occurring along both the horizontal and vertical axes of the brain's architecture (Pessoa, 2022).

Horizontal integration refers to communication between regions at the same hierarchical level (e.g., cortico-cortical pathways). Vertical integration, on the other hand, connects different levels of the nervous system (cortex, subcortex, and brainstem), linking cognition with fundamental processes such as emotion and autonomic state. Crucially, ITMA proposes that the specific configuration of this connectivity—be it horizontal, vertical, or mixed—emerges directly from the quality and combination of the sensorimotor, cognitive, and socio-affective inputs of the training experience, which dictate how the brain reorganizes itself to sustain metastability.

Furthermore, ITMA recognizes that functional segregation is as fundamental as integration. Metastability, as the ideal state of brain functioning, does not emerge merely from more connected networks, but rather from the dynamic equilibrium between coupling and decoupling, between synchronization and autonomy. Thus, martial arts training should simultaneously modulate horizontal and vertical integration as well as functional segregation, promoting a brain architecture capable of responding with flexibility, coordination, and specialization to environmental demands.

### 5.4 Embodied inputs: the negentropic sources of training

In the embodied aspect, ITMA proposes that training interventions can be organized into three broad categories, which often overlap and provide the essential negentropic inputs for neural reorganization.

First, there are interventions that involve movement with additional cognitive tasks—a form of motor-cognitive dual-task training where the practitioner “thinks while moving” (Herold et al., 2018). Examples include conscious and deliberate movements, which require the practitioner to maintain rhythmic and continuous patterns while simultaneously directing attentional focus to internal body sensations (interoception), monitoring their own mind-wandering, and re-establishing focus whenever necessary (Russell and Arcuri, 2015). The negentropic input here is the rich integration of motor control, interoceptive feedback, and attentional regulation.

Second, there are interventions that involve movement with embedded cognitive tasks, in which thinking and acting occur in an integrated and simultaneous manner (Herold et al., 2018). Practices such as grappling, striking, armed combat, or equestrian activities exemplify this category (Russo and Ottoboni, 2019; Ohtani et al., 2017). The negentropic input derives from the complex dynamic integration between automatic motor processes and strategic cognitive control. Although repeated practice leads to automation, cognitive control does not disappear; it remains crucial, requiring the continuous use of executive functions—such as working memory, inhibitory control, and cognitive flexibility—to adapt to the constant information, distractions, and changes from the environment or an opponent (Christensen et al., 2019; Diamond and Ling, 2016; Diamond, 2000; Leisman et al., 2016).

Third, training experiences can also include specific voluntary behaviors and contemplative practices that directly modulate physiology. This involves the use of postures, vocalizations, and especially controlled breathing, as well as practices like chanting, meditation, or ritualistic dances present in some traditions. The negentropic input, in this case, is the direct modulation of autonomic activity, achieved mainly by increasing the inhibitory influence of the vagus nerve on the heart (Porges, 2011, 2021).

### 5.5 Social and environmental inputs: the central role of interactions

Beyond embodied inputs, ITMA emphasizes the central role of social and environmental inputs in training experiences. The relational nature of these practices is not a mere background context but an active element in inducing patterns of neural synchronization and, consequently, in modulating functional connectivity.

For human beings, social connections are not optional; they constitute a fundamental biological imperative for the species' survival and development (Porges, 2022). Social separation and isolation compromise virtually all aspects of human development (Cacioppo and Cacioppo, 2014), and can manifest as impairments in the domains of brain health (Porges and Furman, 2011). Evolutionarily, the human brain was shaped as a social organ, specialized in receiving, processing, and transmitting information to and from other brains around it (Cozolino, 2013, 2019; Adolphs, 2009). (Cozolino 2014) termed the space between individuals, filled with this flow of visible and invisible information, the “social synapse”.

Three central neurobiological mechanisms allow social experiences to influence functional connectivity. The first is non-verbal bio-behavioral synchronization, which refers to the often unconscious attunement of gestures, postures, rhythms, and vocalizations between individuals. This mutual harmonization sustains positive resonance—a shared sense of connection, safety, and wellbeing (Vacharkulksemsuk and Fredrickson, 2012; Fredrickson, 2013). Neurophysiologically, this process is regulated by the social engagement system, a neural network that integrates five cranial nerves (V, VII, IX, X, and XI) responsible for controlling facial expression, voice intonation, and listening. The activation of this system is what enables physiological co-regulation: the ability of one individual, through social interaction, to influence and calm the physiological state of another, creating a virtuous cycle of safety and connection (Porges, 2009, 2022). The second is the mirror neuron system, which allows for motor and empathetic learning through observation and imitation (Rizzolatti and Sinigaglia, 2010). The third is the theory of mind, the capacity to infer the intentions and mental states of others, which is fundamental for strategic interaction (Frith and Frith, 2005).

ITMA proposes that martial arts intensively train all these mechanisms in multiple ways. This occurs through the instructor-student relationship, which can serve as a secure base of attachment and alloparental care for children (Bowlby, 1988; Hrdy, 2009); through co-regulation among practitioners themselves (Kok et al., 2013; Schore, 2003); and through the creation of a culture of belonging and emotional safety (Cacioppo and Patrick, 2008). Combat itself is a rich input, experienced as rough-and-tumble play (distinct from violence) and as an intense exercise of theory of mind (Mickelsson and Stylin, 2021; StGeorge and Fletcher, 2020; Kimmel and Rogler, 2019). Additionally, positive physical touch or contact during training stimulates the release of oxytocin (Tarsha and Narváez, 2023; Rassovsky et al., 2019), while collective rituals and symbolic gestures strengthen social bonds (Clapton and Hiskey, 2020; Collins, 2005; Donnelly and Kidd, 2021; Lindenberg et al., 2011). The relational scope can even extend to interspecies co-regulation, as occurs in equestrian modalities (Porges, 2021).

Finally, the physical-sensory environment acts as another major modulator. According to the theory of enactive cognition, the brain does not passively process an external world but actively constructs meaning through continuous interaction between perception and action. The training space, therefore, is not a passive backdrop but a dynamic ecosystem of affordances—perceived opportunities for action that emerge from the relationship between the physical environment and the practitioner's perceptual-motor capacities. These environmental inputs, by modulating the flow of sensorimotor information, directly influence neural synchronization states and contribute to the processes of experience-dependent synaptic plasticity (Varela et al., 1991).

In sum, ITMA postulates that the rich tapestry of inputs—embodied, social, and environmental—that constitute the martial arts training experience does not act upon a passive brain. On the contrary, these flows of information are continuously integrated with the brain's own intrinsic activity. It is from this synergistic fusion of the internal and external worlds that neural dynamics are shaped, moment to moment, driving the processes of synchronization and plasticity that ultimately sustain metastability and guide the system toward quasicriticality.

### 5.6 Preliminary empirical evidence

Although ITMA is an emerging theoretical proposal, it already finds support in a growing body of preliminary empirical evidence (summarized in Table 1) pointing toward the modulation of functional connectivity and the optimization of metastability. These changes are reflected in multidimensional patterns of neural reorganization, involving not only horizontal and vertical integration but also functional segregation. As detailed in Table 1, the disparate findings from various martial arts studies can be interpreted through ITMA's lens as converging toward these core mechanisms of neural reorganization.

**Table 1:** Summary of preliminary empirical studies supporting ITMA's core mechanisms.

**Study (reference)**	**Martial art**	**Population**	**Main finding (observed data)**	**ITMA interpretation (theoretical proposition)**
(Tao et al. 2016)	Tai Chi Chuan and Baduanjin	Elderly	Increased resting-state functional connectivity between the medial prefrontal cortex and the hippocampus	Represents a mixed pattern of vertical integration (limbic-cortical) and horizontal coordination within higher-order cognitive systems.
(Kim et al. 2015)	Taekwondo	Children	Enhanced brain connectivity from the cerebellum to the frontal and parietal cortices.	A robust instance of vertical integration supporting motor-cognitive coordination.
(Berti et al. 2019); (Duru and Balcioglu 2018)	Karate	Elite karate practitioners	Stronger subcortical-cortical connectivity and increased integration across sensorimotor, visual, and executive networks.	Consistent with a pattern involving both horizontal and vertical optimization.
(Rassovsky et al. 2019); (Harwood-Gross et al. 2020)	Jujitsu/General	At-risk youth and adults	Martial arts practice, especially tasks involving high tactile contact (e.g., grappling), leads to a significant increase in salivary oxytocin release.	ITMA postulates that oxytocin, as a neuromodulator, influences functional connectivity. Its association with executive functions suggests that the relational inputs of training are markers of improved vertical integration between the central autonomic network and executive cortical systems.

The table differentiates between the key findings as reported in the original studies (Observed Data) and their conceptual interpretation within the Integrative Theory of Martial Arts (ITMA Proposition).

In the domain of embodied inputs, several studies across different martial arts have reported enhanced connectivity patterns. (Tao et al. 2016) observed greater integration between the medial prefrontal cortex and the hippocampus in elderly practitioners of tai chi chuan and baduanjin. According to ITMA, this finding can be interpreted as a mixed pattern, combining vertical integration (between limbic and cortical structures) with horizontal coordination within higher-order cognitive systems. (Kim et al. 2015) documented enhanced connectivity between the cerebellum and frontal and parietal cortices in children practicing taekwondo. ITMA proposes that this pattern represents a robust instance of vertical integration supporting motor-cognitive coordination. (Berti et al. 2019) and (Duru and Balcioglu 2018) identified stronger subcortical-cortical connectivity and increased integration across sensorimotor, visual, and executive networks in karate practitioners. This finding is consistent with ITMA's proposition of a pattern involving both horizontal and vertical optimization.

In the relational domain, preliminary evidence also supports ITMA's core mechanisms. (Rassovsky et al. 2019) and (Harwood-Gross et al. 2020, 2021) demonstrated that martial arts practice contributes to the endogenous release of oxytocin, which has been associated with enhanced processing speed and inhibitory control. ITMA postulates that these functions can be considered markers of improved vertical integration between the central autonomic network and executive cortical systems (Benarroch, 1993; Thayer et al., 2012; Yee et al., 2016). These changes reinforce the notion that social-affective inputs are powerful modulators of functional connectivity.

Together, these preliminary findings (Table 1) suggest that different martial arts practices—through their specific training regimes—can induce unique, but converging, patterns of neural reorganization. ITMA proposes that the dynamic interplay between integration and segregation is precisely what collectively sustains the metastable dynamics necessary for healthy neural functioning and cognitive flexibility, allowing for flexible switching between different functional brain states.

### 5.7 Optimizing the signal-to-noise ratio: the *sine qua non* condition

The mere practice of martial arts, in itself, does not guarantee the optimization of functional connectivity nor the induction of the desired brain dynamics. For the mechanisms of synaptic plasticity to be effectively triggered, ITMA postulates that the pattern of neural synchronization must exhibit a high signal-to-noise ratio (SNR). In this context, the “signal” refers to the coherent, relevant, and task-specific neural information, while the “noise” comprises all activity that interferes with this signal, whether of external origin (such as irrelevant environmental stimuli) or internal origin (such as physiological states of threat or danger to life, fatigue, inflammation).

Training with a low SNR—where the brain is overloaded with noise or the signal is diffuse—can result in maladaptive plasticity, or it may simply fail to generate any lasting change. Therefore, optimizing the SNR is a *sine qua non* condition for training to induce functional neural changes toward quasicriticality (Harris and Thiele, 2011).

It is important to highlight that the principles of neuroplasticity have been progressively elucidated and consolidated in the scientific literature (e.g., Kleim and Jones, 2008; Merzenich et al., 2014; Nahum et al., 2013; Maier et al., 2019; Kolb and Muhammad, 2014; Keshavan and Eack, 2019). These principles provide the instructor with a solid theoretical-practical framework for the intentional design of training experiences, allowing them to structure sessions that maximize the relevant signal, minimize interfering noise, and thereby increase the neuroplastic efficacy of the practice. The instructor, in this scenario, acts as a “neurosculptor,” capable of shaping the practitioners' neural dynamics through a pedagogy or andragogy that is evidence-based and guided by a fundamental neurophysiological principle: the signal-to-noise ratio.

### 5.8 Inter-individual variability: the rule, not the exception

ITMA postulates that response variability among individuals is not an exception but the natural rule when working with complex systems like the human brain. This variability is a direct consequence of three fundamental characteristics of neural dynamics: chaos (small differences in initial states lead to large differences in outcomes); non-linearity (the relationship between stimulus and response is not proportional, such that large stimuli can generate small responses, while small stimuli can trigger significant changes); and non-ergodicity (group averages do not apply to individuals). For these reasons, plasticity is an intrinsically individual process, with each brain responding uniquely to the same training stimuli (Kelso, 1995; Molenaar, 2004; Voss et al., 2017).

This phenomenon of inter-individual response variability (Herold et al., 2021)—known in the context of martial arts as the Kreese-Miyagi Effect (Hackney, 2013)—can be categorized into different states of responsiveness: responders (positive outcomes), super-responders (above-expected outcomes), non-responders (no significant change), and adverse responders (negative outcomes). These states emerge from the complex interaction between endogenous and exogenous factors (Herold et al., 2019).

Endogenous factors are the unique initial conditions of each practitioner. Age and biological sex modulate the magnitude and type of plastic change, with replicable, behaviorally relevant baseline differences in functional brain dynamics associated with sex (Merzenich et al., 2014; Nahum et al., 2013; Ryali et al., 2024). The levels of neuromodulators (e.g., GABA, acetylcholine, dopamine) are also crucial, directly influencing the neuroplastic potential, attention, and motivation of the practitioner (Heba et al., 2015; Voss et al., 2017; Merzenich et al., 2014). An individual's baseline inflammatory status is a critical factor, as chronic inflammation can reflect a metabolically “stuck” state—a blocked salugenesis—that impairs the system's overall resilience and capacity for adaptive reorganization (Naviaux, 2018, 2023, 2014). Genetic polymorphisms—such as the 7-repeat allele of *DRD4* and the short allele of *5-HTTLPR*—have been associated with greater plasticity and sensitivity to environmental influences, suggesting that practitioners with these variants may be particularly responsive to martial arts training experiences (Pluess and Belsky, 2013). Physiological and behavioral characteristics—such as high cortisol reactivity, higher cardiac vagal tone, and high sensory processing sensitivity—may also indicate greater neuroplastic potential (Pluess and Belsky, 2013).

Many of these endogenous factors seem to converge on the modulation of emotional processing, a key function of the amygdala. Amygdala activity correlates with physiological reactivity and neuroticism—the tendency to experience negative emotions (Deng et al., 2019; Norris et al., 2007). This suggests that prior assessments of neuroticism and other traits that modulate physiological reactivity (e.g., hypnotic capacity, repressive coping see Wickramasekera (1988)) can provide valuable insights for interpreting practitioner response variability. The centrality of the amygdala can be explained by its dual function: although known for processing threats, evidence shows that it responds even more robustly to positive stimuli (Sergerie et al., 2008). This sensitivity to both emotional extremes suggests that amygdala reactivity may be a central mechanism that helps explain inter-individual response variability (Pluess and Belsky, 2013).

Exogenous factors, in turn, explain variability based on the program itself. The main one is the way in which the principles of neuroplasticity are operationalized, that is, the instructor's ability to design experiences with a high signal-to-noise ratio to optimize connectivity and metastability. Second, the training environment itself—encompassing its physical characteristics (affordances) and social context (e.g., the degree of emotional safety)—is a critical exogenous factor that shapes the practitioner's neurophysiological state and ability to learn from the training session (Porges, 2011). Third, the quality of implementation is fundamental; poorly executed programs can compromise results, leading to what is known as a Type III Error, which invalidates the analysis of the intervention's effects (Barry, 2019; Durlak, 2016; Proctor et al., 2010).

### 5.9 Implications of neuroplasticity for martial arts

The understanding of neuroplasticity, as articulated by ITMA, generates crucial implications that redefine practice, research, and evaluation in the field of martial arts.

**Experience design as a critical factor:** ITMA opposes the dominant view that the benefits of martial arts practice are automatic or inherent to a specific style (Hartmann and Kwauk, 2011). Crucial for neuroplasticity is the intentional design of the experience. This includes the quality of practice, personalization to individual needs, and even the strategic design of activities to optimize the release of neuromodulators at opportune moments, maximizing plastic potential. The focus shifts from the modality to the design.

**The Instructor as a neurosculptor:** the role of the martial arts instructor is elevated from a mere technical transmitter to an architect of neuroplastic experiences (Foisy et al., 2020; Baldini et al., 2014). Their mastery of the principles of neuroplasticity and their ability to manage the signal-to-noise ratio can be direct predictors of a program's effectiveness.

**Variability, risk, and ethics:** response variability is the rule. It is crucial that researchers and instructors consider the endogenous and exogenous factors that lead to different states of responsiveness (including adverse responses). This has a direct ethical implication: neuroplasticity is a neutral mechanism. Poorly planned or implemented programs can be harmful, inducing maladaptive plasticity and worsening functional connectivity instead of improving it.

**Baseline assessment for personalization and analysis:** if variability is the rule, a direct implication is the need for baseline assessments. Mapping the practitioner's initial state—by evaluating personality traits (such as neuroticism), physiological reactivity, etc.—serves a crucial dual function. Proactively, it informs the personalization of interventions, allowing the instructor to adjust the training design to optimize the signal-to-noise ratio for that individual and anticipate adverse responses. Retrospectively, this initial assessment helps explain the inter-individual variability of outcomes observed at the end of the program, conferring greater interpretive power to research and practice.

**Evaluation of implementation and emergent outcomes:** the evaluation of a program must be twofold. It is necessary not only to measure the outcomes in practitioners but also, separately, the quality of program implementation based on implementation science criteria. Furthermore, one must recognize that outcomes are a mix of planned and emergent aspects that arise spontaneously from the system's self-organization. This requires flexibility from the instructor and researcher to acknowledge and value the unpredictable.

## 6 Assessment of outcomes: a new generation of neurophysiological metrics

### 6.1 Assessing individual trajectories: the *N* = 1 longitudinal design

ITMA proposes a rupture with evaluation models based on group averages. Given the non-ergodic, non-linear, and chaotic nature of the brain, the trajectories of neurophysiological change are, by definition, unique and idiosyncratic. Therefore, the single-case (*N* = 1) longitudinal design emerges as the new methodological frontier for capturing, with high temporal resolution, how each nervous system responds to martial arts training interventions (Molenaar, 2004; Kelso, 1995; Fisher et al., 2018; Molenaar and Campbell, 2009; Krasny-Pacini and Evans, 2018).

### 6.2 The functional triad: measuring quasicriticality

To quantitatively assess quasicriticality, ITMA proposes a shift from static and linear indicators to dynamic and non-linear metrics. In this context, a functional triad based on heart rate variability (HRV) is adopted as an accessible and ecologically valid proxy for the underlying neurophysiological dynamics, reflecting the system's efficiency, adaptability, and resilience (Shaffer and Ginsberg, 2017; Goldberger et al., 2002; Shaffer et al., 2014; Thayer and Lane, 2000; Thayer et al., 2009; Porges, 2021, 2011; Laborde et al., 2018; Ernst, 2017). The three complementary indicators that compose the functional triad are DFA α1, SampEn, and SD2/SD1.

**DFA** **α1 (detrended fluctuation analysis):** this indicator quantifies the short-term fractal correlations in an HRV time series, revealing the degree of long-range dependence between successive RR intervals (Peng et al., 1995). A value close to 1.0 indicates a “pink noise” (1/f noise) dynamic—a signature of healthy and efficient functioning in self-organized complex systems (Goldberger et al., 2002). Significant deviations toward higher values (excessive persistence and rigidity) indicate subcritical tendencies, while lower values (randomness, loss of correlation) indicate an approach toward a supercritical regime, both of which are considered suboptimal.

**SampEn (Sample Entropy):** This measures the irregularity and unpredictability of the HRV time series (Richman and Moorman, 2000). A high SampEn indicates a system with high adaptive complexity, capable of flexibly responding to a variety of environmental and physiological challenges (Pincus and Goldberger, 1994). Conversely, low SampEn values reflect rigidity, excessive predictability, and a reduced capacity for adaptation—a pattern frequently observed in conditions of autonomic dysfunction or in states of disease (Beckers et al., 2005).

**SD2/SD1 ratio (Poincaré plot):** The SD2/SD1 ratio, derived from the geometry of the Poincaré Plot, quantifies the balance between long-term (SD2) and short-term (SD1) heart rate variability (Brennan et al., 2001). A value greater than 1.0 reflects a system capable of absorbing rapid perturbations without compromising its long-term stability, thus serving as a marker of physiological resilience (Borghesi et al., 2024).

Together, these metrics offer an integrated assessment of the system's proximity to the quasicritical regime and can be complemented by other HRV indicators for a richer analysis.

A critical—and often counter-intuitive—aspect of the approach proposed by ITMA is that the success of a martial arts training program is not necessarily defined by an absolute increase in the individual values of these indices, as is often assumed in traditional interventions based on linear biomarkers. Instead, the primary goal is to guide the system into the so-called “quasicritical zone”, characterized by a dynamic equilibrium between order and chaos, where high complexity, adaptive efficiency, and resilience to perturbations coexist (Cocchi et al., 2017; Goldberger et al., 2002). More importantly, two dynamic criteria must be evaluated beyond simply “reaching the optimal zone”: the system's ability to maintain this state over time (temporal stability)—reflecting neurophysiological sustainability (Beckers et al., 2005); and the speed and efficiency of returning to the quasicritical state after controlled or natural perturbations (dynamic resilience)—reflecting the system's allostatic capacity (Buchheit, 2014). Therefore, the assessment of success, according to ITMA, must focus on three interconnected dimensions: (1) reaching the quasicritical zone; (2) the ability to maintain this condition over time (temporal stability); and (3) the speed and efficiency of return after perturbations (dynamic resilience).

### 6.3 Mixed methods and the value of qualitative analysis

ITMA suggests a mixed-methods approach, recognizing that quantitative data extracted from HRV metrics capture only one dimension of the neurophysiological change process. Incorporating qualitative methods allows contextualizing individual trajectories of change, offering a richer and more ecologically valid understanding of the psychosocial, affective, and relational processes that influence plasticity and resilience (Creswell and Plano Clark, 2017; Fetters et al., 2013). This integration makes it possible to identify mediating and moderating factors that may not be detected by physiological measures alone, as well as to broaden the understanding of the subjective mechanisms that underpin the changes observed in quantitative indicators (Onwuegbuzie and Leech, 2006). Thus, ITMA aligns with a growing movement in health science that emphasizes the consilience between objective and subjective data as a fundamental strategy for capturing the complexity of human phenomena (Palinkas et al., 2011).

### 6.4 From idiographic data to public policy: a multilevel framework for translational science

ITMA advances translational science by proposing a multilevel framework capable of transforming idiographic findings into population-based public policies without losing individual data integrity. This approach seeks to overcome the ecological fallacy—the methodological error of inferring individual conclusions from group averages (Robinson, 1950)—and aligns with the emerging paradigms of precision medicine, idiographic psychology, and translational neuroscience based on complex dynamics. The proposed framework integrates three complementary stages:

**Stage 1—Bayesian meta-analyses of** ***N*** **=** **1 studies:** the first stage involves the statistical aggregation of *N* = 1 longitudinal studies through Bayesian meta-analyses. This approach combines idiographic data without losing the uniqueness of each individual trajectory, being more sensitive to heterogeneity and enabling probabilistic inferences about population effects, with variability treated as primary data rather than as noise to be controlled (McElreath, 2020).

**Stage 2—agent-based modeling (ABM):** the second stage utilizes ABM, a methodology from complex systems science for the large-scale simulation of interactions among thousands of individual agents (Bonabeau, 2002). In the ITMA context, each “agent” represents a practitioner with a specific neurophysiological profile. Based on parameters from the *N* = 1 studies, ABM models can predict how different intervention scenarios would affect the population, considering emergent effects from interactions among individuals (Macy and Willer, 2002).

**Stage 3—pragmatic trials with heterogeneity preservation:** the third stage proposes pragmatic trials (Thorpe et al., 2009), designed to maximize ecological realism and external generalization by maintaining the natural diversity of contexts and participants. This means avoiding methodological filters that exclude participants with comorbidities, different age groups, or baseline profiles. ITMA suggests statistical methods sensitive to variability, such as hierarchical or Bayesian models, and recommends a heterogeneity-preserving trial design (Glasgow et al., 2003). Furthermore, the integration of individual metrics (*N* = 1) within the group trial allows tracking of individual and population effects.

In summary, by articulating Bayesian meta-analyses, agent-based modeling, and pragmatic trials with heterogeneity preservation, ITMA proposes a robust translational cycle connecting science, clinical practice, and public policies, respecting the complexity of living systems and human diversity (Torous et al., 2017).

## 7 Discussion

Throughout history, martial arts have served as more than methods of combat—they have functioned as vehicles for health, education, spiritual growth, and cultural continuity (ICM, 2021; Bowman, 2019). ITMA uptades and expands these roles through a unifying scientific lens, proposing that carefully designed martial training experiences can optimize brain health by inducing the nervous system into dynamic states of quasicriticality—a dynamic regime of neural activity that supports health, efficient information processing, adaptability, and resilience. By integrating findings from diverse disciplines under a mechanistic and multiscale framework, ITMA offers a unified model to understand and potentiate martial arts training effects on neurophysiological systems.

It is crucial to position ITMA correctly: at its core, this article presents a foundational theoretical framework, not a complete translational protocol. The primary objective was to establish the plausibility and coherence of the mechanistic model—the “map”—before detailing the specific “routes” of its application. However, the theory's high translational potential is one of its most promising features. Because it is grounded in first principles of complex systems science, ITMA is not restricted to martial arts. They serve as a rich and ecologically valid “exemplar model”, but the underlying framework may, in the future, be adapted and extended to investigate other interventions.

The implications of ITMA transcend the individual. As quasicriticality exhibits fractal properties (Peng et al., 1995; Goldberger et al., 2002), with patterns of health and wellbeing repeating across scales from brains to ecosystems (Lent, 2021), a martial arts program can act as a vector of sustainable development. By applying global principles to local contexts—a process of glocalization (Baker, 2022)—these practices demonstrate that neither a person's biography nor a society's history defines their destiny (Dweck, 2006; Goleman, 2005), in this way, promoting hope and resilience.

### 7.1 Limitations

As with any emergent theoretical model, ITMA must be subjected to rigorous empirical testing. While it offers a unified, mechanistic, and multiscale explanation of how martial arts may optimize brain health, its fundamental mechanisms and predictive capacity remain, at this stage, theoretical. Future studies will need to verify whether the training variables proposed—particularly the embodied, social and environmental inputs—are indeed capable of inducing, sustaining, and re-establishing the neurophysiological dynamics described.

The preliminary empirical evidence reviewed herein, while promising, was not generated within the conceptual framework of ITMA. These studies suggest that martial arts modulate functional connectivity, but none explicitly evaluated whether such changes converge toward the quasicritical regime. Therefore, empirical studies are needed to test the core propositions of ITMA using non-linear neurophysiological metrics, longitudinal single-case (*N* = 1) designs, and mixed-methods approaches that combine dynamic quantitative assessment with qualitative contextualization.

Furthermore, a core methodological proposition of ITMA is the use of HRV metrics as a proxy for brain quasicriticality. While this remains an indirect measurement, this limitation represents a deliberate methodological trade-off made to prioritize accessibility and ecological validity. The high cost and logistical constraints of neuroimaging methods limit research to laboratory settings. In contrast, the accessibility and low cost of wearable HRV technology are precisely what make the proposed *N* = 1 longitudinal designs and real-world pragmatic trials feasible, democratizing research and practice beyond specialized labs. This approach allows data to be collected in the actual training environments where martial arts are taught and practiced. Nevertheless, a crucial frontier for future research, as noted, is to empirically validate the degree to which this practical proxy correlates with direct markers of quasicritical brain dynamics.

Also, while ITMA proposes a translational framework from idiographic data to public health policy, no full implementation of this multilevel strategy has yet been undertaken. Its practical application—from *N* = 1 Bayesian aggregation to agent-based modeling and heterogeneity-preserving pragmatic trials—remains a fertile field for future interdisciplinary collaboration.

A further consideration is the innovative and demanding nature of the proposed methodological framework. Successfully implementing the full suite of tools—from longitudinal *N* = 1 data collection to agent-based modeling—will likely require the formation of interdisciplinary teams that bridge expertise in martial arts pedagogy, neurophysiology, and advanced computational statistics. This highlights a forward-looking challenge for the field: to foster the collaborative networks and develop the shared tools necessary to advance this line of inquiry.

### 7.2 Future directions

These limitations, however, also illuminate the frontiers of inquiry that ITMA opens, guiding a robust agenda for future investigation. To operationalize the validation and expansion of ITMA, a multiphase research roadmap is proposed, progressing from foundational and methodological questions to large-scale translational applications.

**Phase 1**—**foundational validation and methodological refinement:** the first critical step is to validate the theory's core tools and assumptions. Research in this phase should focus on proxy validation and correlation with functional domains. It will be necessary to (1) conduct studies to correlate the proposed HRV functional triad (DFA α1, SampEn, SD2/SD1) with direct neuroimaging markers of quasicriticality and metastability from fMRI and EEG. This step is essential to confirm the proposed biomarkers as robust proxies for the desired brain dynamics; and (2) implement *N* = 1 longitudinal designs to investigate the degree to which changes in the validated biomarkers correlate with measurable improvements across the domains of brain health (cognitive, sensory, social-emotional, behavioral, and motor).

**Phase 2—mechanistic exploration and personalization:** once the measurement tools are validated, research can delve deeper into the mechanisms and inter-individual variability. Studies can design experiments to map how specific training “ingredients”—such as particular embodied, social and environmental inputs—produce specific outcomes within the different domains of brain health. For example, which combinations of tasks and neuroplasticity principles are most effective at enhancing attentional control (cognitive domain) or emotional regulation (social-emotional domain)? The ultimate goal is to understand how these input-outcome relationships are mediated by the proposed neurophysiological cascade, thereby identifying the most potent combinations for driving the system toward metastability and quasicriticality. Then, studies can conduct baseline assessments of individual factors to identify which factors best predict responsiveness to ITMA-based interventions. This is key for developing personalized and adaptive protocols.

**Phase 3—translational science and public health application:** with a deeper understanding of mechanisms and personalization, the focus shifts to real-world application. Studies can implement heterogeneity-preserving pragmatic trials to test the effectiveness and sustainability of programs designed with ITMA principles in real-world settings, such as communities, schools, hospitals, or workplaces. Then, utilize data from *N* = 1 and pragmatic trials to inform agent-based simulation models (ABM) to predict the large-scale public health impacts of implementing ITMA-based programs. Finally, investigate how idiographic metrics (*N* = 1) can be integrated into large-scale public health monitoring systems, addressing the challenge of applying personalized insights at a population level.

ITMA, therefore, inaugurates a new paradigm where tradition and innovation meet, providing new criteria for designing interventions and new tools for their evaluation. It offers a powerful framework for instructors, researchers, health professionals and policymakers to align martial arts with the deeper goals of public health and sustainable human development. From this perspective, martial arts—when guided by scientific intentionality—become instruments of individual and collective transformation—capable of shaping healthier brains, strengthening communities, and contributing to a more abundant and prosperous future for all.

## Data Availability

The original contributions presented in the study are included in the article/supplementary material, further inquiries can be directed to the corresponding author.
